# Light-Promoted Rhodopsin Expression and Starvation Survival in the Marine Dinoflagellate *Oxyrrhis marina*

**DOI:** 10.1371/journal.pone.0114941

**Published:** 2014-12-15

**Authors:** Zhiling Guo, Huan Zhang, Senjie Lin

**Affiliations:** 1 Department of Marine Sciences, University of Connecticut, Groton, Connecticut 06340, United States of America; 2 Key Laboratory of Tropical Marine Bio-resources and Ecology, South China Sea Institute of Oceanology, Chinese Academy of Sciences, Guangzhou, China; 3 Department of Environmental Science, Ocean University of China, Qingdao, Shandong 266100, China; 4 State Key Laboratory of Marine Environmental Science, Xiamen University, Xiamen, Fujian 361005, China; King Abdullah University of Science and Technology, Saudi Arabia

## Abstract

The discovery of microbial rhodopsins in marine proteobacteria changed the dogma that photosynthesis is the only pathway to use the solar energy for biological utilization in the marine environment. Although homologs of these rhodopsins have been identified in dinoflagellates, the diversity of the encoding genes and their physiological roles remain unexplored. As an initial step toward addressing the gap, we conducted high-throughput transcriptome sequencing on *Oxyrrhis marina* to retrieve rhodopsin transcripts, rapid amplification of cDNA ends to isolate full-length cDNAs of dominant representatives, and quantitative reverse-transcription PCR to investigate their expression under varying conditions. Our phylogenetic analyses showed that *O*. *marina* contained both the proton-pumping type (PR) and sensory type (SR) rhodopsins, and the transcriptome data showed that the PR type dominated over the SR type. We compared rhodopsin gene expression for cultures kept under light: dark cycle and continuous darkness in a time course of 24 days without feeding. Although both types of rhodopsin were expressed under the two conditions, the expression levels of PR were much higher than SR, consistent with the transcriptomic data. Furthermore, relative to cultures kept in the dark, rhodopsin expression levels and cell survival rate were both higher in cultures grown in the light. This is the first report of light-dependent promotion of starvation survival and concomitant promotion of PR expression in a eukaryote. While direct evidence needs to come from functional test on rhodopsins *in vitro* or gene knockout/knockdown experiments, our results suggest that the proton-pumping rhodopsin might be responsible for the light-enhanced survival of *O*. *marina*, as previously demonstrated in bacteria.

## Introduction

Chlorophyll-based photosynthesis was conventionally considered as the only way to use the solar energy for biological utilization in marine ecosystems until the discovery of microbial rhodopsins in marine microbes which can harvest light energy nonphotosynthetically [1], [2]. Similar to animal rhodopsins which work as light sensor [3]–[6], microbial rhodopsins also consist of seven transmembrane α-helices that form an internal pocket, in which a photoreactive chromophore, retinal, is attached through a Schiff base linkage to a lysine residue to absorb the light energy [7]–[10]. Upon illumination, the retinal undergoes isomerization and induces conformational changes in the associated rhodopsin, which in turn initiates the ion transport or photosensory functions [2], [11].

Microbial rhodopsins were firstly discovered in the halophile archaeum *Halobacterium salinarum* in the form of the light-driven proton pump bacteriorhodopsin [8]. Subsequently, three additional types of microbial rhodopsins were discovered in *H*. *halobium*: a chloride uptake pump [12] and two phototaxis receptors [13]–[15]. Homologs of these rhodopsins were later identified in various microorganisms including eubacteria (e.g. α-proteobacteria, γ-proteobacteria, and cyanobacteria) and eukaryotes (e.g. fungi and algae). The potential functions of these rhodopsins range from the light-driven proton (e.g. bacteriorhodopsin, proteorhodopsin, and xanthorhodopsin) or chloride ion pumps (halorhodopsin) to phototaxis receptors (sensory rhodopsin and phoborhodopsin) [16]–[21]. For instance, the first microbial rhodopsin-encoding gene in the ocean was discovered from γ-proteobacteria of the SAR86 group, and the encoded protein, proteorhodopsin, was shown to function as a light-driven proton pump [1]. Recently, proteorhodopsins-encoding genes were found in a diverse array of Bacteria and Archaea inhabiting the ocean's photic zone [2], and even bacteria on terrestrial plant leaves [22], suggesting that the utilization of light energy via proton-pumps is widely adopted in the microbial world. These proton-pumping rhodopsins generate ATP through the use of the electrochemical membrane potential produced by the translocated protons, thereby powering general cellular metabolism or energy-requiring functions such as flagellar rotation, nutrients transport, or acidification of cellular compartments [10], [18], [23]–[25]. Some microbes harbor more than one type of rhodopsins. In addition to the multiple forms of rhodopsins in *H*. *halobium* mentioned above, the halophile *Salinibacter ruber* has four different microbial rhodopsins, including two sensory rhodopsins, one halorhodopsin, and one xanthorhodopsin [21], [26], [27]. The sensory rhodopsins in halophilic archaea function as light sensors to direct cells to favorable (orange) light while away from unfavorable (ultraviolet) light, while in marine bacterioplankton, they function as the daytime sensor or depth gauge [2], [23], [28], [29]. The chloride ion- pumping rhodopsins in halophilic archaea can be used in the regulation of ionic content and osmotic state [2].

Homologs of rhodopsin have been reported from various eukaryotic algae, including the chlorophytes *Chlamydomonas reinhardtii* and *Acetabularia acetabulum*, the cryptophytes *Guillardia theta* and *Cryptomonas* sp., the glaucophyte *Cyanophora paradoxa*, the euglenophyte *Euglena gracilis*, and various species of dinoflagellates [30]–[35]. In *C*. *reinhardtii*, experiments have shown that channelrhodopsin-1 is a combined photoreceptor and light-gated ion channel that induce the photomotile behavior of the alga [36], and two structurally distinct sensory rhodopsins (CSRA and CSRB) mediate phototaxis to low- and high- intensity light, respectively [34], [37]. The functions of rhodopsins in other algae remain to be demonstrated, although gene sequence homology analysis has shown that most of them belong to the sensory type except in dinoflagellates, in which most rhodopsin sequences resemble the proton-pumping rhodopsins in bacteria [33]. Among dinoflagellates, *Oxyrrhis marina* is exceptional in that it appears to harbor both the sensory type and the proton-pump type rhodopsins [38], [39].

*O. marina* is a widespread and ecologically important marine heterotrophic dinoflagellate [40]. It has been considered as an unusual basal branch of the dinoflagellate lineage, rendering it a target for the study of the evolution patterns and genome organization within the crown group Alveolata [41]. Due to its global distribution, ease of isolation and maintenance, *O. marina* has been used as an important model organism for a broad range of ecological and ecophysiological studies [41], [42]. *O*. *marina* culture under light condition displays a pinkish color in high concentration, suggesting a possibility of expression of high abundance of rhodopsins [43]. In addition, it exhibits phototaxis to light, suggesting its photosensory ability [44]. However, the associations of these light-dependent activities with the different forms of rhodopsins have not been investigated. Besides, most of the previously reported *O. marina* sensory rhodopsin sequences were not from the axenic cultures and were not full-length, therefore the posibility that some of the sequences were from the bacterial or food algal sources could not be completely excluded, thereby casting some question about the existance of how many types of rhodopsin in this species.

In this study, we conducted an in-depth search for rhodopsin sequences by generating two dinoflagellate spliced leader (DinoSL)-based cDNA libraries of *O*. *marina* and sequencing them using Illumina technology. Based on the rhodopsin sequences resulting from the transcriptome along with those previously reported, we designed specific primers for the new forms of rhodopsins identified, and retrieved their full-length cDNAs. We then quantified their expression levels under both normal light: dark cycle (LD) and continuous dark (DD) conditions using reverse-transcription quantitative PCR (RT-qPCR).

## Methods

### Algal culture

Both *O*. *marina* (CCMP1795) and the prey alga *Dunaliella tertiolecta* (CCMP1320) were purchased from the Provasoli-Guillard National Collection Center of Marine Phytoplankton in West Boothby Harbor, Maine, USA. *O*. *marina* was grown in autoclaved, 0.22 µm-filtered seawater supplied with *D. tertiolecta* as food. *D*. *tertiolecta* was maintained in f/2-Si medium [salinity 28 practical salinity units (PSU), pH 8.0] at 18°C on a 14:10 h light: dark cycle at a photon flux of ∼100 µ E m^−2^ s^−1^. *O*. *marina* cells in the exponential growth phase were collected for experiments.

### Illumina sequencing of the DinoSL-based transcriptomes

*O*. *marina* cultures were starved for five days and then fed with *D*. *tertiolecta* (5×10^5^ cells•mL^−1^, ∼10 prey cells per *O*. *marina*). Samples were collected 1 h before and 12 h after feeding; at both time points very few prey alga cells were microscopically detectable in the culture. The cells were harvested by centrifugation in 50-mL tubes at 3000×g at cultural temperature (18°C) for 10 min, and were transferred into 1.5-mL tubes after removing the supernatant and pelleted by centrifugation at 15,000×g for 1 min. The cell pellets were immediately re-suspended in Trizol reagent (Invitrogen, Carlsbad, CA, USA) and kept at −80°C until RNA extraction.

RNA was isolated and purified using the Direct-zol RNA MiniPrep kit (Zymo Research Corp., Orange, CA) following the reported method [45], and eluted in 30 µL diethylpyrocarbonate (DEPC)-treated, sterilized ddH_2_O. The quality of RNA was assessed using RNA gel electrophoresis and the quantity was measured using a NanoDrop ND-1000 spectrophotometer (Thermo Scientific, Wilmington, DE, USA).

To obtain the better coverage of the whole transcriptomes, the first-strand cDNAs were synthesized with the isolated RNA as templates using two different primers: a modified oligo (dT) primer 454BT7dT, and a modified random primer 454BT7N9 (where N is any of the 4 nucleotides) adopted from Moreno-Paz and Parro [46] with modification to facilitate 454 sequencing. All the primers used in this study are shown in Table 1. To exclude the interference of prey alga and bacteria in the transcriptomic data analysis, we prepared PCR-amplified *O. marina* cDNAs using the dinoflagellate mRNA-specific DinoSL as the 5′-end primer pairing with 454BT7 as the 3′-end primer, a method proven to be effective to specifically amplify dinoflagellate cDNAs [33], [47], [48]. PCR was performed with the following program: 94°C for 1 min, 5 cycles of 95°C for 20 s, 72°C for 2.5 min, 5 cycles of 95°C for 20 s, 65°C for 30 s, 72°C for 2 min, 5 cycles of 95°C for 20 s, 60°C for 30 s, 72°C for 2 min, and 15 cycles of 95°C for 20 s, 58°C for 30 s, and 72°C for 2 min, and an additional elongation step of 7 min at 72°C. Two amplified libraries were mixed (454BT7dT-based cDNAs: 454BT7N9-based cDNAs  = 2∶1), electrophoresed in an agarose gel, and the 400–2000 bp size fractions were recovered as previously reported [38]. The two resulting cDNA libraries (from samples collected 1 h before and 12 h after feeding, respectively) were sheared and subjected to one lane of Illumina sequencing at the Sequencing Lab of National Center for Genome Resources (Santa Fe, NM, USA). After Illumina sequencing, the reads from the two samples were pooled for assembly and annotation. Due to the relatively short insert size and the long adaptor sequence (52 bp) in the primers used to synthesize the cDNAs, many reads after trimming the adaptors and primers were too short and had to be excluded from further analysis. The cDNA fragments homologous to rhodopsins sequences were identified from the annotation results.

**Table 1:** Primers used in this study.

Primer name	Primer sequence (5′-3′)	Application	Source
454BT7dT	GAGACTATGCGCCTTGCCAGCCCGCTCAGTA	*O*. *marina* cDNAs synthesis	This study
	ATACGACTCACTATAGGGAGT(16)V		
454BT7N9	GAGACTATGCGCCTTGCCAGCCCGCTCAGTA	*O*. *marina* cDNAs synthesis	[66]
	ATACGACTCACTATAGGGAGNNNNNNNNN		
454BT7	GAGACTATGCGCCTTGCCAGCCCGCTCAGTA	Amplification of the *O*. *marina* cDNA libraries	This study
	ATACGACTCACTATAGGGAG		
OxyrhoS1F1	AGTCAGCAGTCCAAGAACAAGAAGT	*O*. *marina* SR1 3′ RACE	This study
OxyrhoS1R1	CCAGGACCAGAATGAAGAACACCAT	*O*. *marina* SR1 5′ RACE	This study
OxyrhoS1R2	GCCAGGTCCAGCAACAGCAA	*O*. *marina* SR1 5′ RACE	This study
OxyrhoS2F1	CCGAAGGCTAAGCAGTTCAACTACAC	*O*. *marina* SR2 3′ RACE	This study
OxyrhoS2R1	CAGGCTGCTCAACAAGTCCTCATC	*O*. *marina* SR2 5′ RACE	This study
OxyrhoS2R2	GCACACCAGTCGTCAAGAAGAACA	*O*. *marina* SR2 5′ RACE	This study
OxyrhoS3F1	CCTGCTCATTGTTCTGGACATTCTTATG	*O*. *marina* SR3 3′ RACE	This study
OxyrhoS3R1	GCTGGCAGAGCAGGTAGAACAA	*O*. *marina* SR3 5′ RACE	This study
OxyrhoS3R2	CCATCTTGGCAGCCTTGTCATCC	*O*. *marina* SR3 5′ RACE	This study
DinoSL	NCCGTAGCCATTTTGGCTCAAG	5′ Racer	[38]
Modified T7	TAATACGACTCACTATAGGGAG	3′ Racer	This study
Oxy18SNF1	GCTTGTCACATAGGCGATTATATTCTTACC	*O*. *marina 18S rRNA* forward	This study
Oxy18SNR2	GACCTGTTATTGCCCGACTCTTCC	*O*. *marina* 18S rRNA reverse	This study
OxyrhodF	GCCATGGCGCCTTTAACTGGGGAC	*O*. *marina* Proton-pump Type Rhodopsin forward	This study
OxyrhodRa	CTAACGCAGCAGCTTGCCTC	*O*. *marina* Proton-pump Type Rhodopsin reverse	This study
OxyActinF1	CTGCGATGTACGTGCAGATC	*O*. *marina actin* forward	This study
OxyActinR	TAGCACAACTTCTCCTTCACATC	*O*. *marina actin* reverse	This study
OxyCO1F	CTTGCCTGTATTATCTGCTGCTT	*O*. *marina cox1* forward	This study
OxyCO1R	AGTATGCTCTTGTATCTACTTCTAACC	*O*. *marina cox1* reverse	This study
18ScomF1	GCTTGTCTCAAAGATTAAGCCATGC	*D*. *tertiolecta* 18S rRNA forward	[67]
18ScomR1	CACCTACGGAAACCTTGTTACGAC	*D*. *tertiolecta* 18S rRNA reverse	[67]
Oxy18SNF2	GTGACAAGAAATAACTGTGTGTGGCTC	*O*. *marina* 18S rRNA qPCR	This study
Oxy18SNR1	AGCTACTCGCAACACGCTTAACC	*O*. *marina* 18S rRNA qPCR	This study
OxyrhoS1F3	CCGTTGCTGTTGCTGGACCT	*O*. *marina* SR1 qPCR	This study
OxyrhoS2F3	TGTGCTCCAGAAGGCGAACGA	*O*. *marina* SR2 qPCR	This study
OxyrhoS3F3	GGATGGTGCTCTTCGTCATTGTCA	*O*. *marina* SR3 qPCR	This study
OxyRhodF2	CACTACTTCMGNATCTTCAACTC	*O*. *marina* PR qPCR	This study
OxyRhodR	CAGAGGMACRGTCARCARCCARTC	*O*. *marina* PR qPCR	This study
OxyActinF2	ACGTGCAGATCCAGGCTG	*O*. *marina actin* qPCR	This study
Dinocox1F6	GGTTCTTTGGACATCCTGAAGTTTA	*O*. *marina cox1* qPCR	This study
Dun18SF2	GGGTTGTAGCGGTCAGCCTTTG	*D*. *tertiolecta*18S rDNA qPCR	This study
Dun18SR1	AGCCAGAGTCCTATCGTGTTATTCCA	*D*. *tertiolecta*18S rDNA qPCR	This study

### Rapid amplification of rhodopsin cDNA 5′ and 3′ ends (RACE)

Based on the partial rhodopsin-like sequences obtained from Illumina-based transcriptome sequencing and the top hits of the *O. marina* EST sequences in GenBank database, specific primers were designed using Beacon Designer version 3.0 software (Premier Biosoft International, Palo Alto, CA, USA) to obtain the full length rhodopsin cDNAs using the DinoSL-based RACE method [49]. The first-strand, modified oligo (dT)-based cDNAs were used as the template for PCR amplification with *O*. *marina*-specific rhodopsin primers paired with DinoSL or a modified T7 primers. When nested PCR was needed, the first PCR products were diluted 1000-fold and used as the template. Amplicons were excised from the agarose gel, purified using Zymo DNA clean Gel DNA Recovery Kit (Zymo Research, CA, USA) and directly sequenced or cloned into a T-vector as reported [49]. Five to ten clones were randomly picked for sequencing. The 5′ and 3′-end sequences retrieved were assembled to obtain the full-length rhodopsin cDNA sequences.

### Phylogenetic analysis

To examine the relationship between the full-length rhodopsin sequences obtained in this study with the previously reported rhodopsins from other organisms, phylogenetic tree was inferred. We aligned the deduced rhodopsin amino acid sequences with homologs from other representative organisms using CLUSTAL X (version 2.0.11; Gap Opening 60), and then corrected manually [50]. A protein model test was run using MEGA 5.20 to identify the best-fit amino acid substitution model. The best-fit model LG with gamma distribution and empirical frequencies (LG+G+F) was then employed for Maximum Likelihood (ML) analysis using PhyML package [51]. In addition, Neighbor-Joining (NJ) analysis [52] was also run in MEGA 5.20, and Bayesian analysis [53] was run in MrBayes 3.22 with model jumping setting for 500,000 generations (two independent runs were performed and the final standard deviation of split frequencies was 0.01), with trees sampled every 100 cycles and the first 1250 cycles discarded. The reliability of the tree topology was evaluated using bootstrap analysis with 1000 resampling for NJ analysis and 500 for ML analysis.

### Long-term effects of light-dark conditions on *O. marina* growth and survival rates and rhodopsins expression

To investigate the effect of different culture conditions on the growth and survival rates of *O. marina*, and the expression patterns of different rhodopsin genes, the following experiment was carried out. Prior to the experiment, *O*. *marina* was acclimated to experimental prey at food-saturated concentrations (∼10 prey cells per *O*. *marina*) under the LD cycle mentioned above for two weeks, and then starved for seven days until no prey alga was microscopically detectable.

The experimental cultures were set up in triplicates under each condition. On the first day, *O*. *marina* was provided with prey at ∼10 prey cells per *O*. *marina*. One triplicate set was maintained under normal LD condition, and a parallel set was incubated in DD with the culture bottles covered 2 layers of heavy duty aluminum foil then covered with a thick black plastic bag. All the cultures were maintained in a temperature-controlled (18°C) environmental chamber. For the cultures grown in DD, care was taken to avoid exposing the cultures to light. Cultures in both conditions were observed microscopically every day to check whether the prey cells persist. Cell abundance was monitored every two days by microscopic cell counts using a Sedgwick-Rafter chamber as previously reported [54]. Cell size [the length of minor axis (width) and major axis (length)] was measured from 30–40 cells in each sample (∼100 cells in triplicate samples) using NIS-Elements D 4.00.03 (Build 75) Software under a microscope. The cell volume was calculated for all 100 cells as: 4/3×3.14×(length)×(width/2)^2^, and then averaged. Every 2–3 days, 10^5^–10^6^ cells were harvested for RNA extraction as described above. As the negative control, ∼10^6^ prey alga *D. tertiolecta* cells were also harvested.

### Reverse-transcription quantitative PCR (RT-qPCR)

For all the LD and DD samples and the *D. tertiolecta* sample, RNA extraction was carried out as described above. For each sample, ∼100 ng of the total RNA was used as the template to synthesize the first-strand cDNAs using Quantitect Reverse Transcription Kit (Qiagen, Hilden, Germany) that contained an optimized blend of oligo-dT and random primers, with the potential genomic DNA contaminant removed in the process. The cDNAs were diluted 100-fold, and 4 µL of each was used as the template for RT-qPCR using the corresponding gene-specific primer sets.

Seven *O. marina* genes were analyzed for the expression pattern detection in both LD and DD samples using RT-qPCR, including four rhodopsin genes (one proton-pumping, three distinct sensory types), and three non-rhodopsin genes which served as the potential references to normalize the expression of rhodopsin genes: 18S rRNA, *actin* and *cox1*. For each gene analyzed, a large cDNA fragment was PCR-amplified and used as the standard DNA to construct the standard curve. PCR was performed using the first-strand cDNAs as the template with the corresponding primer sets: OxyrhodF-Ra for proton pump rhodopsin; OxyrhoS1F1-R1, OxyrhoS2F1-R1, and OxyrhoS3F1-R1 for sensory rhodopsins SR1, SR2, and SR3, respectively; Oxy18SNF1-NR2 for 18S rRNA; OxyActinF1-R for *actin*; OxyCO1F-R for *cox1* under the same program as described previously [49] except the amplification of *cox1* in which the annealing temperature was 55°C. PCR amplicons generated were purified using Zymo DNA clean Gel DNA Recovery Kit and checked by electrophoresis to ensure the absence of any aberrant or degraded products. The concentrations of the purified amplicons were measured and a dilution series of the standard DNA equivalent to 10^2^, 10^3^, 10^4^, 10^5^ and 10^6^ molecules per 4 µL were made for each gene analyzed in qPCR as reported [49].

RT-qPCR was carried out with Bio-Rad iQ SYBR Green Supermix kit (Bio-Rad Laboratories, Hercules, CA, USA) on an Applied Biosystems StepOnePlus Real-time PCR detection system (ABI, Carlsbad, CA) at the condition of 1 cycle of 10 min at 95°C, 40 cycles of 15 s of denaturation at 95°C, and 1 min of annealing/extension at 60°C (for *cox1*, 30 s/30s anneal/extension). An additional dissociation step was added to generate a melting-curve thermal profile to confirm the amplification of single PCR product with the expected melting profile. In each reaction, 4 µL of cDNAs, 0.5 µL of each primer (at a final concentration of 5 µM) and 5 µL of SYBR Green Supermix were added. The qPCR assays were performed in triplicates for each experimental sample and duplicate for each standard sample, with a no-template negative control included in each run. Based on the correlation between the threshold cycle number (C_t_) and the logarithmic-transformed cDNA copy number established from the dilution series of the standard DNA, transcript copy number for each sample was calculated automatically by the system based on its C_t_.

The relative expression stability of these three potential reference genes was analyzed using geNorm (http://ikmbio.csie.ncku.edu.tw/GN/), which indicated that the expression of 18S rRNA and *cox1* was more stable; these two genes were then selected as reference genes.

### Statistical analyses

All the data were shown as mean±standard deviation. Differences between treatments in cell concentration, cell volume, the total RNA content, and rhodopsin expression level were tested by student's *t*-test in software PASW 18.0. One-way analysis of variance (ANOVA) based on LSD (equal group size) or Bonferroni (unequal group size) was performed to compare the data on different days. For all tests, values of *p*<0.05 were considered as the criterion for statistical significance.

## Results

### Illumina transcriptome data of *O*. *marina*

**Figure 1:**
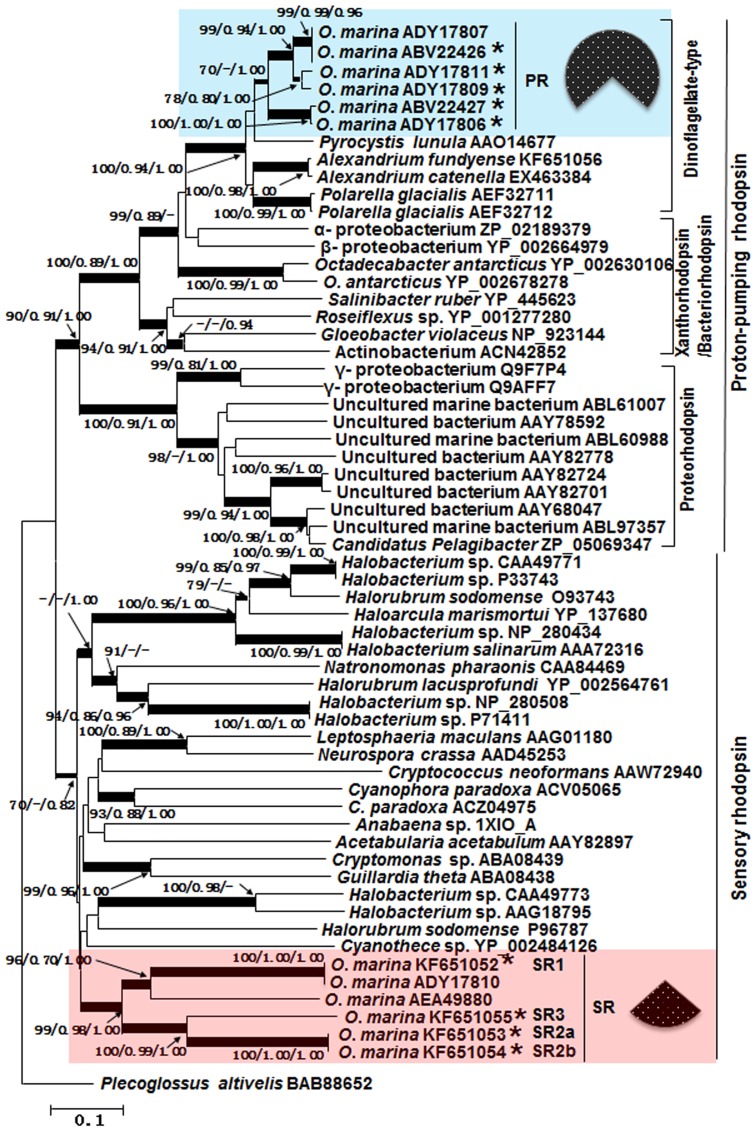
Phylogenetic tree inferred from rhodopsin amino acid sequences. Shown at nodes are bootstrap values from Neighbor-Joining (left), SH-like value of maximum likelihood (medium) and Bayesian analysis (right); only values ≥70%/0.7 at critical nodes are shown. The thickest branches denote bootstrap values of>90%, medium-thick branches values of 70 to 90%, and thin branches values of <70%. The scale bar indicates the substitutions rate per nucleotide. Tree is rooted with *Plecoglossus altivelis*. The two major types, the proton-pumping (PR) and the sensory type (SR), are indicated on the far right while subgroups within PR are shown next. Pie slices indicate the relative transcript abundances of the two major types (with subtypes within each type combined) in terms of the percentage of reads. Asterisks depict sequences obtained in this study that were either new or matched previously reported sequences (accession numbers indicated).

Clustered at 90% identity and 30 bp overlap criteria using ABySS software, the combined transcriptome dataset yielded 2,883 contigs, with the length ranging from 150 to 1411 bp, 1,342 of which had hit at E-value <10^−3^ to the documented genes in public databases, 64% of which hit dinoflagellates or their alveolate relatives (*Perkinsus marinus*, apicomplexan and ciliates) (S1 Figure), with *P. marinus* and *O*. *marina* as the top hits (S2 Figure). Within the 1,342 annotated contigs, we found 19 distinct rhodopsin-like sequences, 16 of which showed high identity (mostly 97%–100%) to the previously reported putative proton-pumping type (e.g. ABV22426, ADY17811), or putative sensory type rhodopsin sequences (ADY17810) of *O*. *marina* in GenBank, while the other 3 shared only 35–64% amino acid identity to the existing *O*. *marina* rhodopsin sequences. The rhodopsin contigs we obtained were represented by a total of 255,487 reads. After normalization to the sequencing scale and gene length, the Reads Per Kilobase Per Million (RPKM) values were 11,988 for the proton-pump rhodopsin-like sequences (PR) and 4,857 for the sensory rhodopsin-like sequences (SR), which accounted for 71% and 29%, respectively (Fig. 1).

### Assembly of full-length sensory rhodopsin cDNA sequences

Besides those that matched previously reported dinoflagellate rhodopsins, we found three distinct, potentially novel forms of rhodopsin. We designed specific primers and used RACE to obtain their full-length coding region sequences. Three sequences (named as SR1, SR2, and SR3) were retrieved. We found that there are two variants in the SR2 type, which had identical coding region yet different 5′ non-coding region (named SR2a and SR2b). The open reading frames of these four genes were 756 bp, 762 bp, 762 bp and 741 bp, respectively. SR1 shared 99% amino acid identity with the reported rhodopsin (ADY17810), while SR2 and SR3 shared only 38% or less amino acid identity with any reported rhodopsins. Furthermore, the conserved amino acid residues of the reported *O*. *marina* PR type (ADY17811) rhodopsin were either absent (e.g. YNALSFGIAAMGSATVFFWLQL in position 17–38, YRTALTITGIVTWIATYHYFRIFNSWV in position 49–75) or different (e.g. many of the 22 retinal binding residues) in these three sequences (S1 Table).

### Phylogenetic relationships of rhodopsin sequences

We aligned the deduced amino acid sequences of the new rhodopsins with the representative homologs from *O*. *marina* and other microbial organisms reported in GenBank. The sequences were clustered into two distinct groups, the proton-pumping and the sensory type clades (Fig. 1). In the proton-pumping group, the sequences from *O*. *marina* and other dinoflagellates (*Pyrocystis lunula*, *Polarella glacialis*, *Alexandrium catenella*, and *A*. *fundyense*) formed a monophyletic subgroup, and grouped with proteorhodopsins (PRs) and xanthorhodopsins/bacteriorhodopsin. The newly discovered rhodopsin sequences fell within the sensory rhodopsin group. SR1 (KF651052) was closest to *O*. *marina* rhodopsin sequence reported previously (ADY17810) [39], while SR2a (KF651053)/SR2b (KF651054) and SR3 (KF651055) formed well-separated groups, indicating that these were new among the diverse forms of sensory rhodopsins in *O. marina*. The proton-pumping *O. marina* rhodopsin sequences also exhibited substantial sequence variations (Fig. 1).

### Growth, cell volume, and RNA content of starved *O. marina* under light and dark conditions

**Figure 2:**
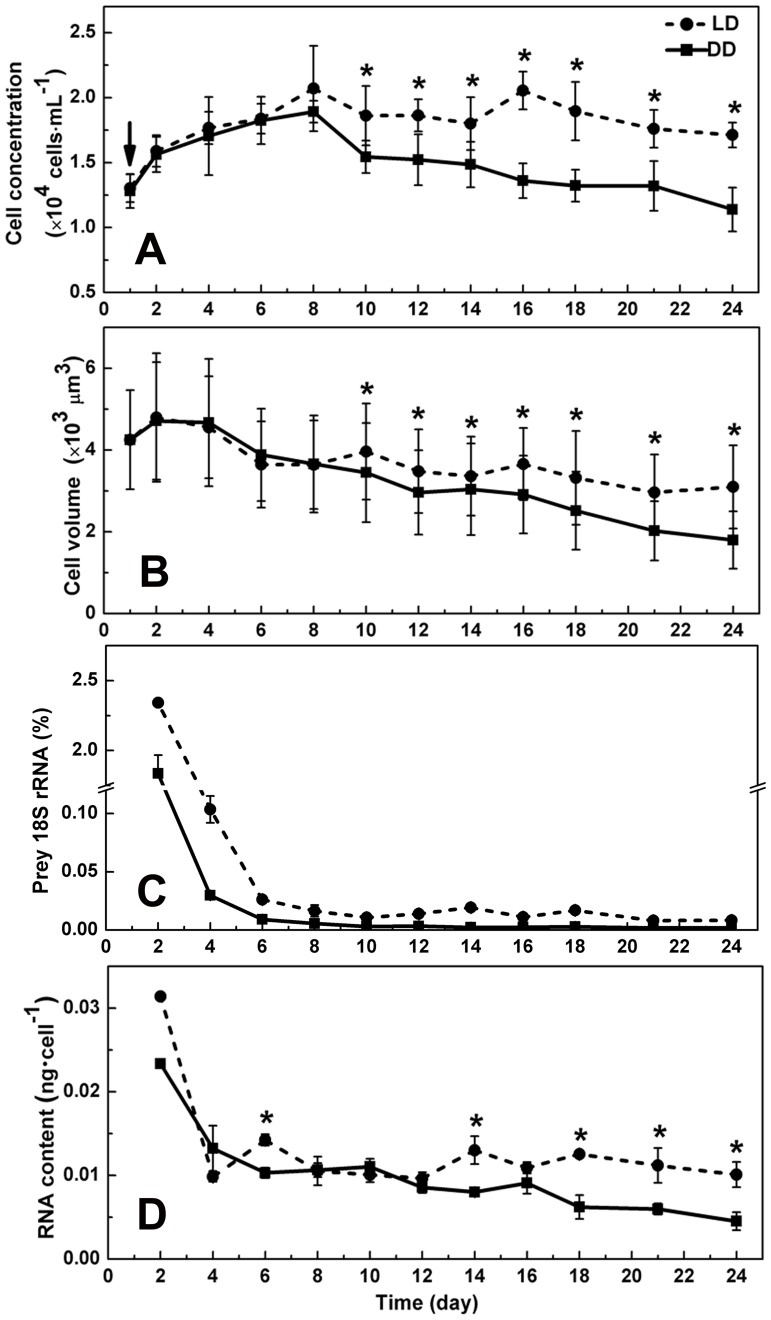
Growth curve (A), cell volume (B), prey 18S rRNA percentage (C), cellular total RNA content (D) of starved *O*. *marina*. RT-qPCR was carried out to quantify the 18S rRNA level of both *O*. *marina* and *D*. *tertiolecta* in the cDNA libraries from both conditions. Y-axis in (C) indicates the ratio of prey 18S rRNA and *O*. *marina* 18S rRNA. LD, cultures grown under light: dark cycle; DD, cultures grown under continuous darkness; error bars, standard deviation. Significant differences between LD and DD groups were marked with “*”. Arrow in the figure denotes time when food was supplied.

Different growth patterns were observed between LD and DD treatments, both without resupply of prey since the one-time supply on day 1 (Fig. 2A). Statistical analyses showed that the cell concentration in LD group increased significantly in the first 8 days (*p*<0.05; compared with day 1), stayed stable from day 8 to day 18 (*p*>0.05; compared with day 8), and then decreased from day 21 (*p*<0.05; compared with day 8). In DD group it increased from day 1 to day 8 (*p*<0.05; compared with day 1), and then decreased until the last day (*p*<0.05; compared with day 8). The DD group had the same growth rate (0.034 d^−1^) with that in LD in the first 8 days, and then stayed at lower cell concentrations than the LD group until the last day (*p*<0.05). In a repeated experiment, a similar pattern was observed that cell concentration in the DD group stayed lower than that in the LD group after day 8, although the temporal trend (rate of decline) of the cell population exhibited a slight difference, which may be attributed to different physiological states of the cultures (S3 Figure).

Cell volume in both LD and DD groups started to decrease the sixth day after the initial feeding (Day 1; *p*<0.05), and LD cultures showed slower decrease in general, and the difference between the LD and DD treatments increased over time (Fig. 2B). From day 10 and afterward, cell volume in DD group stayed lower than that in the LD group.

In both treatments, the cell concentration of the prey alga *D*. *tertiolecta* dropped to less than 1% of *O. marina* on day 2. No prey cells were noticeable both in the cultures and inside *O*. *marina* cells since the fourth day. However, there was a possibility that the RNA from *D. tertiolecta* ingested by *O. marina* would remain intact in the first several days. To assess the potential impact of RNA from *D*. *tertiolecta* on the measurement of *O. marina* RNA, we used RT-qPCR to quantify the 18S rRNA level of *D*. *tertiolecta* in the cDNA libraries from both conditions using *D*. *tertiolecta*-specific 18S rRNA primers. We found that the *D*. *tertiolecta* 18S rRNA copy number was less than 2.5% of that of *O. marina* counterpart in all samples (Fig. 2C), indicating that the contribution of *D*. *tertiolecta* RNA to the total RNA from both LD and DD samples was negligible.

Generally, the total RNA per *O. marina* cell in LD group remained at a relatively stable level (∼0.012 ng/cell; *p*>0.05), while in DD group, it decreased from the fourth day (*p*<0.05) and was lower than that in the LD group most of the time in the 24 day period (Fig. 2D), indicating cells in DD condition were less active in gene expression.

### Expression levels of rhodopsin under starvation

We investigated the expression levels of different rhodopsin genes (PR, SR1, SR2 and SR3) for *O*. *marina* cultured in both conditions using RT-qPCR. We first verified the specificity of the primers designed. The electrophoresis analysis of the resultant qPCR amplicons showed that a single band for each primer set was produced. The melt curve analysis showed that a single melt peak occurred for each primer set. The PCR efficiency and correlation coefficient in qPCR were>80% and>0.999, respectively. All these indicated that the primer sets for different genes were highly specific and showed high PCR efficiency. No amplification was detected for prey alga *D*. *tertiolecta* cDNA when *O. marina* primer sets were used in RT-qPCR.

When normalized to the amount of the total RNA used in qPCR, the expression level of *actin* in both conditions increased during the time course (S4-A Figure; *p*<0.05), while the 18S rRNA and *cox1* transcription in both conditions remained at a relatively stable level throughout all growth stages (S4-B Figure, S4-C Figure). This and the geNorm analysis result all suggested that *actin* (stability index = 1.2) was not a good reference gene. Therefore, only 18S rRNA (stability index = 0.681) and *cox1* (stability index = 0.671) were used as reference genes to normalize the expression level of rhodopsin genes.

**Figure 3:**
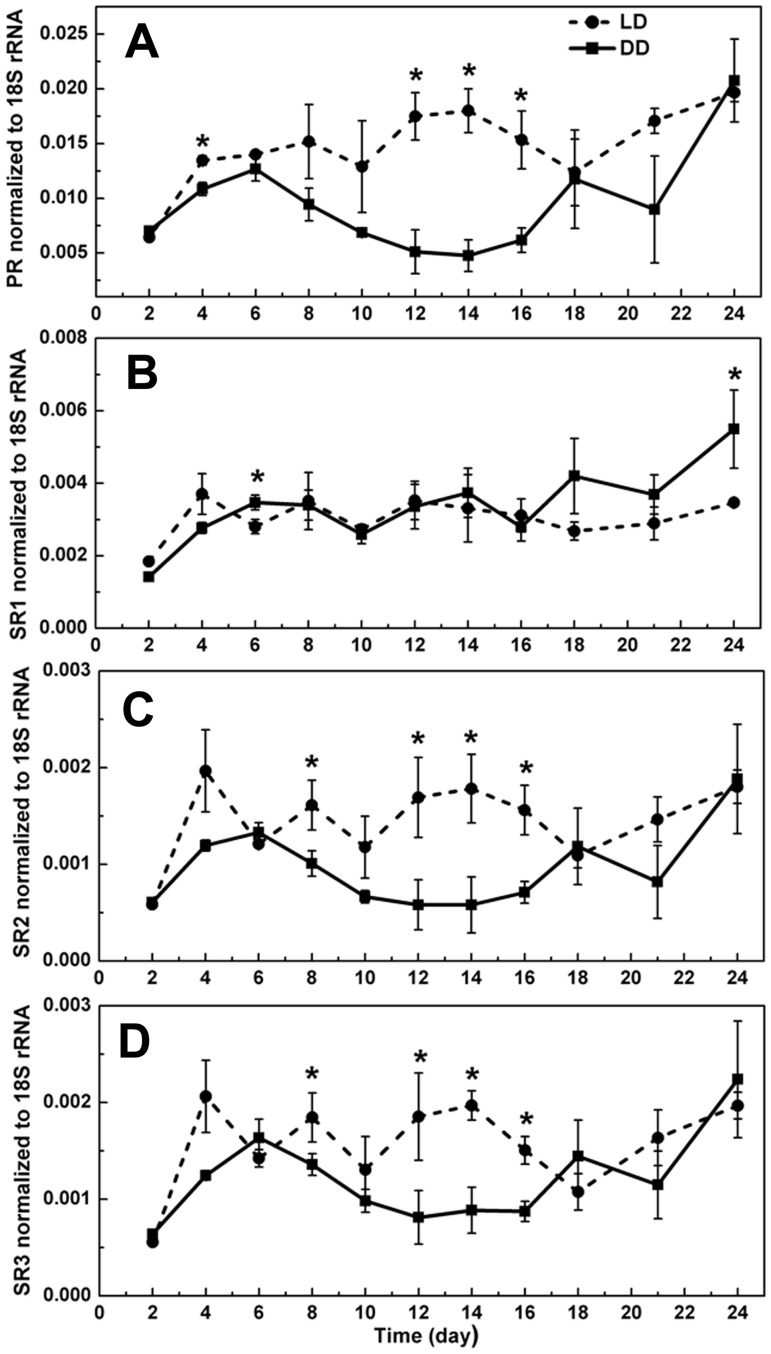
Expression levels of rhodopsin genes as normalized to 18S rRNA cDNA. LD, cultures grown under light: dark cycle; DD, cultures grown under continuous darkness; error bars, standard deviation. (A) PR; (B) SR1; (C) SR2; (D) SR3. Significant differences between LD and DD groups were marked with “*”.

**Figure 4:**
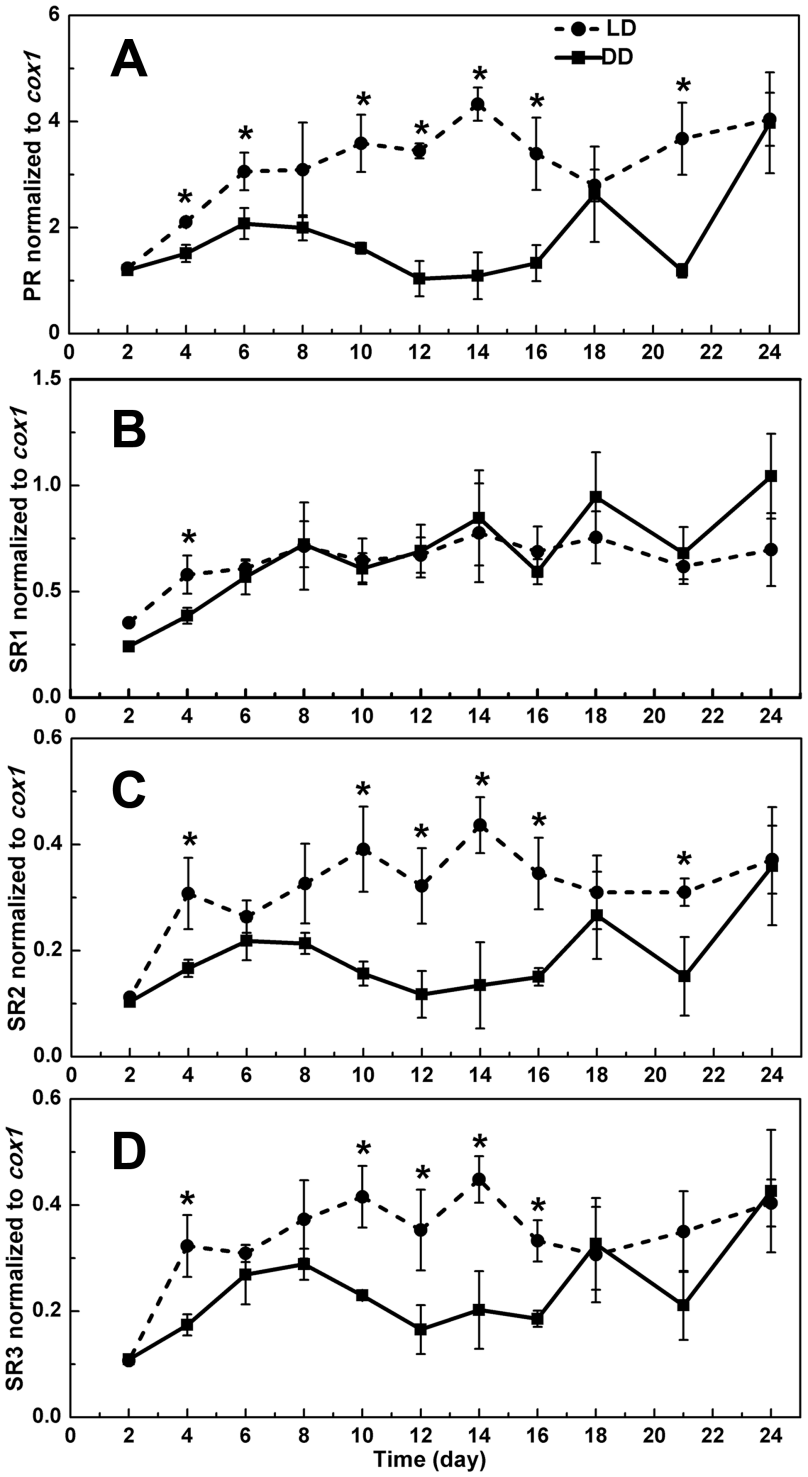
Expression levels of rhodopsin genes as normalized to *cox1* cDNA. LD, cultures grown under light: dark cycle; DD, cultures grown under continuous darkness; error bars, standard deviation. (A) PR; (B) SR1; (C) SR2; (D) SR3. Significant differences between LD and DD groups were marked with “*”.

Regardless which reference gene was used, similar trends of rhodopsin gene transcription were observed (Fig. 3, 4). Among the four rhodopsin genes, the temporal trends in the expression of PR, SR2 and SR3 were similar. Under the LD condition, the expression level of these three rhodopsin genes showed a general increase from day 1 to day 14, and a decrease from day 14 to day 18, and then it slightly increased again. For the cultures in DD treatment, the transcript level of the three genes increased in the first 6 days, and decreased from day 6 to day 12, and then began to increase till the last day. The transcript abundances of these three rhodopsin genes in LD were generally higher than those in DD. Exception occurred toward the end of the 24-d study period, when the difference between the LD and DD cultures started to disappear, likely a result of feedback to the prolonged starvation and over-stress of cells in both LD and DD groups. However, the expression profile of SR1 differed from that of other three genes, and the transcript level did not show significant difference between the two treatments (*p*>0.05).

**Figure 5:**
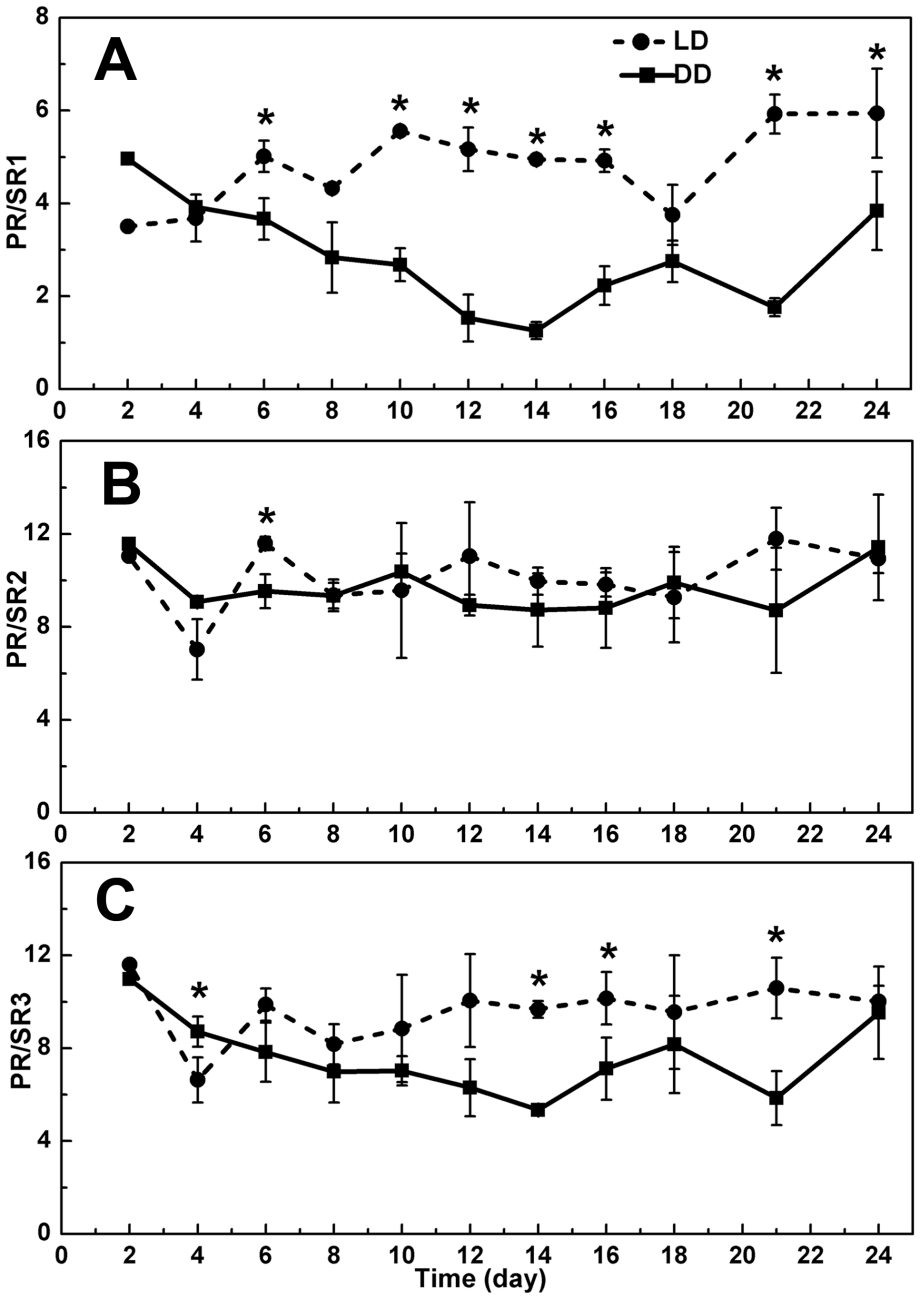
Ratio of expression levels between proton-pump rhodopsin (PR) and sensory rhodopsins (SR1, SR2 and SR3). LD, cultures grown under light: dark cycle; DD, cultures grown under continuous darkness; error bars, standard deviation. (A) PR/SR1; (B) PR/SR2; (C) PR/SR3. Significant differences between LD and DD groups were marked with “*”.

PR exhibited the highest expression levels among all the rhodopsin genes we examined throughout the experimental period. Under LD condition the expression level of this gene can reach up to 6, 12.2, and 11.6 times as high as SR1, SR2, and SR3, respectively, while 5, 11.5, and 11 times under DD condition (Fig. 5).

## Discussion

### *O. marina* origin of transcriptome and rhodopsin sequences

A technical issue associated with molecular genetic studies on heterotrophic organisms is the presence of contaminating prey organisms, which can confound downstream analyses. DinoSL is a specific mRNA marker found at the 5′ end of nucleus-encoded mRNAs in dinoflagellates [38]. This unique spliced leader makes separation of dinoflagellate transcripts from assemblage of different organisms possible [47], [48], [55]. In this study, although the starting RNA derived from a nonaxenic culture, most of our *O. marina* transcriptome sequences showed high similarity to the reported genes from dinoflagellates or their alveolate relatives, verifying again that cDNA libraries prepared using DinoSL as the selective 5′-primer were highly dinoflagellate-specific. A small portion of the sequences did not have dinoflagellate or alveolate organisms as BLAST top hits likely because no homologs have been reported in these organisms. The result indicates that all the rhodopsin sequences obtained from the *O. marina* transcriptome, although some of which differed considerably from each other, were from *O. marina* instead of the prey alga. This was further verified by the recovery of the full-length rhodopsin cDNAs using DinoSL as the forward primer.

### Diverse variants of rhodopsin

PR-encoding genes are ubiquitous in the microbial world, and their functions and physiological and ecological roles have been demonstrated for some bacteria or unidentified picoplankton. For example, Martinez et al. (2007) identified two genetically distinct PRs-containing clones by functionally screening large-insert DNA libraries derived from marine picoplankton, and verified their function as light-activated proton pump [24]. Light-enhanced growth rates and cell yields have been observed in PRs-containing marine flavobacterium *Dokdonia* sp. strain MED134 [56]. The ability of PRs to absorb light energy and converts it to ATP through phosphorylation has been demonstrated in a heterologous host cells exposed to light [24], [57]. Sineshchekov et al. (2002) identified two different sensory rhodopsins from green alga *C*. *reinhardtii*, and showed that they functioned as low- and high-light-intensity photoaxis receptors [37].

Phylogenetic analysis showed that both the currently recognized categories of microbial rhodopsins exist in *O*. *marina*. One group was phylogenetically related to the sensory rhodopsins in archaea and other algae which are involved in the phototactic response, suggesting that these rhodopsins may function as the phototaxis receptors. The other group together with rhodopsins from other dinoflagellates was clustered with proteorhodopsins from bacteria, indicating that these are putatively proton-pumping type rhodopsins. To date, *O*. *marina* is the only dinoflagellate in which both types of rhodopsin have been identified. Besides, within each type, the existence of substantial sequence variations is also unprecedented in dinoflagellates. Whether the variety of rhodopsins also exist in other dinoflagellates warrant further studies.

Furthermore, there are phylogenetically distinct variants in the sensory rhodopsin family in *O. marina*. Whether there is any functional differentiation among the variants in unclear. Multiple sensory rhodopsin receptors with different spectral properties are known to mediate color-sensitive phototaxis responses, spectral discrimination, and light intensity adaptation in different organisms [6], [28], [37]. Although sensory rhodopsin has been hypothesized to have been acquired from ancestral photosynthetic endosymbiont [39], whether they were acquired independently and what functions they play in *O*. *marina* need to be further studied.

### Light-enhanced survival during starvation: evidence of proton-pumping rhodopsin involvement

When fed, *O. marina* appears to grow independently of light. It exhibited continuous population growth in the first six days after one-time feeding in both conditions. The sole energy source for *O*. *marina* in the dark is the prey alga or bacteria in cultures as reported previously [58], so the equivalent growth rate and concurrent disappearance of the prey in light and dark indicated that feeding in *O*. *marina* is light-independent and grazing and digestion rates are not affected by the light. The association of light with grazing and growth varied in different organisms. For example, the mixotrophic dinoflagellate *Fragilidium subglobosum* could grow well phagotrophically without light [59], while dinoflagellate *Karlodinium veneficum* (formerly *Gymnodinium galatheanum*) and chrysophyte *Dinobryon cylindricum* could not survive in continuous darkness even in the presence of food [60], [61].

When starved, *O*. *marina* survived better in the light than in the dark. In thae dark, *O*. *marina* experienced a continuous mortality; while in the light, larger cell population was sustained, indicating that light enhanced the survival rates of *O*. *marina* during periods of starvation. There are two possibilities. One possibility is kleptoplastidy, in which *O. marina* would retain the plastid of the algal prey to perform temporary photosynthesis. However, most kleptoplastid has been reported to function only for short period (e.g. in the case of *Pfiesteria piscicida*, Rubisco and chlorophyll both decay within 2–3 days [62]). Kleptoplastid has not been reported for *O. marina*. Even if it exists, the possibility that kleptoplastid remained active for almost four weeks is highly unlikely.

The second possibility is that *O. marina* is able to acquire energy from light to sustain survival for a period of time during absence of prey. In support of this, we found that rhodopsin genes were expressed at higher levels in the light than in the dark. In previous work, Gómez-Consarnau et al. (2010) showed that *Vibrio* sp. AND4 displayed substantially improved survival rates during starvation in seawater when exposed to light compared with darkness, and found that PR-mediated phototrophy promoted the survival of bacteria during starvation [63]. PR-based phototrophy also enhanced the growth in flavobacterium *Dokdonia* sp. MED 134 cultures under light [56]. PR expression also has been proposed as a molecular mechanism coping with iron deficiency in diatoms [64] and may be used as a mechanism of survival under osmotic pressure [65].

It is intriguing that *O. marina* has several forms of rhodopsin genes, and out of the four forms we examined, three of them displayed elevated expression in the LD culture than in the DD culture. It is hard to differentiate the relative contributions of these rhodopsins to the light-enhanced survival. However, our transcriptomic and qPCR results clearly and consistently indicated that the transcript abundance of PR was remarkably higher (∼2.5-fold in transcriptome and up to 12.2-fold by qPCR) than SR. By analyzing about 18,000 EST from *O. marina*, Slamovits et al. (2011) also found that the proton-pumping type rhodopsin is the most abundantly expressed type [39]. All these suggest that the proton-pumping type rhodopsin may be the major type operating in *O*. *marina* to promote energy acquisition for survival during starvation, and the SR type likely functions in a different way or play a minor role.

Overall, further studies are needed to obtain direct experimental evidence that the proton-pumping type rhodopsin functions to generate ATP and sustain population of *O. marina* and other dinoflagellates during starvation (or nutrient limitation). Such evidence may rely on expressing the genes in a heterologous system and verifying its light energy converting function, or a RNA interference technique to inhibit rhodopsin gene expression in the host dinoflagellate.

## Supporting Information

S1 Figure **Summarized Top-Hit lineage distribution of the cDNAs recovered from our** ***O*** **.** ***marina*** **transcriptomic data.** The majority of the sequences either hit dinoflagellates or their alveolate relatives (apicomplexans and ciliates) or had no matches (others) in GenBank database.
(TIF)

S2 Figure **Detailed Top-Hit species distribution of the cDNAs recovered from our** ***O*** **.** ***marina*** **transcriptomic data.** The majority of the sequences hit dinoflagellates (blue) or their alveolate relatives, apicomplexans (orange), *Perkinsus marinus* (purple) and ciliates (green), and a small fraction hit unknown or nondinoflagellate organisms (gray).
(TIF)

S3 Figure **Growth curve of starved** ***O*** **.** ***marina*** **in a repeated experiment.** The cultures were kept under regular light:dark cycle (LD) and continuous darkness (DD) for 20 days and cell concentrations were monitored every other day. Error bars, standard deviation. Significant differences between LD and DD groups were marked with “*”. Arrow in the figure denotes time when food was supplied.
(TIF)

S4 Figure **Expression levels of three potentially house-keeping genes as normalized to the amount of total RNA.** LD, cultures grown under light: dark cycle; DD, cultures grown under continuous darkness; error bars, standard deviation. (A) *actin*; (B) 18S rRNA; (C) *cox1*. Significant differences between light and dark group were marked with “*”.
(TIF)

S1 Table **Alignment of PR and SR types of** ***O. marina*** **rhodopsin to indicate similarity and difference.** Asterisks depict identical amino acid residues; dots depict positions where residues are chemically similar, with positions dominated by one residue indicated by double dots. In yellow shade are regions conserved in the PR type but absent in the SR type. In red letters are residues that compose retinal pocket, which are all conserved in the PR type but partially different in the SR type. Triangle indicates position where proton donor (green) and receiver (blue) are expected, which are also conserved in the PR type but not so conserved in the SR type.
(DOC)

## References

[1] Béjà O (2000) Bacterial Rhodopsin:Evidence for a new type of phototrophy in the sea. Science 289:1902–1906. 10988064 10.1126/science.289.5486.1902

[2] Fuhrman JA, Schwalbach MS, Stingl U (2008) Proteorhodopsins an array of physiological roles. Nat Rev Microbiol 6:488–494. 18475306 10.1038/nrmicro1893

[3] Kikukawa T, Tamogami J, Shimono K, Demura M, Nara T, et al**.** (2012) Photo-induced Proton Transfers of Microbial Rhodopsins. In: Rijeka, Croatia, Saha S, editors. Molecular photochemistry-Various aspects. In Tech. pp 89–108.

[4] Litmann BJ, Mitchell DC (1996) Rhodopsin structure and function. In: Greenwich, Lee AG, editors. Rhodopsin and G-Protein Linked Receptors, Part A. In JAI Press. pp 1–32.

[5] Ruiz-Gonzalez MX, Marin I (2004) New insights into the evolutionary history of type 1 rhodopsins. J Mol Evol 58:348–358. 15045490 10.1007/s00239-003-2557-8

[6] Yokoyama S (2000) Molecular evolution of vertebrate visual pigments. Prog Retin Eye Res 19:385–419. 10785616 10.1016/s1350-9462(00)00002-1

[7] Bieszke JA, Braun EL, Bean LE, Kang S, Natvig DO, et al (1999) The *nop-1* gene of *Neurospora crassa* encodes a seven transmembrane helix retinal-binding protein homologous to archaeal rhodopsins. Proc Natl Acad Sci USA 96:8034–8039. 10393943 10.1073/pnas.96.14.8034 PMC22183

[8] Oesterhelt D, Stoeckenius W (1971) Rhodopsin-like protein from the purple membrane of halobacterium halobium. Nat New Biol 233:149–152. 4940442 10.1038/newbio233149a0

[9] Englander JJ, Englander SW (1977) Comparison of bacterial and animal rhodopsins by hydrogen. Nature 265(5595):658–659. 859571 10.1038/265658a0 PMC3438911

[10] Sharma AK, Spudich JL, Doolittle WF (2006) Microbial rhodopsins:functional versatility and genetic mobility. Trends Microbiol 14:463–469. 17008099 10.1016/j.tim.2006.09.006

[11] Béjà O, Spudich EN, Spudich JL, Leclerc M, DeLong EF (2001) Proteorhodopsin phototrophy in the ocean. Nature 411:786–789. 11459054 10.1038/35081051

[12] Schobert B, Lanyi JK (1982) Halorhodopsin is a light–driven chloride pump. J Biol Chem 257:10306–10313. 7107607

[13] Bogomolni RA, Spudich JL (1982) Identification of a third rhodopsin-like pigment in phototactic Halobacterium halobium. Proc Natl Acad Sci USA 79:6250–6254. 6959114 10.1073/pnas.79.20.6250 PMC347098

[14] Ihara K, Umemura T, Katagiri I, Kitajima-Ihara T, Sugiyama Y, et al (1999) Evolution of the archaeal rhodopsins:evolution rate changes by gene duplication and functional differentiation. J Mol Biol 285:163–174. 9878396 10.1006/jmbi.1998.2286

[15] Tomioka H, Takahashi T, Kamo N, Kobatake Y (1986) Flash spectrophotometric identification of a fourth rhodopsin–like pigment in *Halobacterium halobium* . Biochem Biophys Res Commun 139:389–395. 3767969 10.1016/s0006-291x(86)80003-1

[16] Graul RC, Sadée W (1997) Evolutionary relationships among proteins probed by an iterative neighborhood cluster analysis (INCA). Alignment of bacteriorhodopsins with the yeast sequence YRO_2_ . Pharmaceut Res 14:1533–1541. 10.1023/a:1012166015402 9434271

[17] Idnurm A, Howlett BJ (2001) Characterization of an opsin gene from the ascomycete *Leptosphaeria maculans* . Genome 44:167–171. 11341726 10.1139/g00-113

[18] Waschuk SA, Bezerra AG, Shi L, Brown LS (2005) *Leptosphaeria rhodopsin*: bacteriorhodopsin–like proton pump from a eukaryote. Proc Natl Acad Sci USA 102:6879–6883. 15860584 10.1073/pnas.0409659102 PMC1100770

[19] Yutin N, Koonin EV (2012) Proteorhodopsin genes in giant viruses. Yutin Koonin Biol Direct 7:34. 23036091 10.1186/1745-6150-7-34 PMC3500653

[20] Finkel OM, Beja O, Belkin S (2013) Global abundance of microbial rhodopsins. ISME J. 7:448–451. 23051692 10.1038/ismej.2012.112 PMC3554412

[21] Balashov SP, Imasheva ES, Boichenko VA, Antón J, Wang JM, et al (2005) Xanthorhodopsin: a proton pump with a light-harvesting carotenoid antenna. Science 309:2061–2064. 16179480 10.1126/science.1118046 PMC3065861

[22] Atamna–Ismaeel N, Finkel OM, Glaser F, Sharon I, Schneider R, et al (2012) Microbial rhodopsins on leave surfaces of terrestrial plants. Environ Microbiol 14:140–146. 21883799 10.1111/j.1462-2920.2011.02554.x PMC3608849

[23] Spudich JL, Yang C, Jung K-H, Spudich EN (2000;Retinylidene proteins structures and functions from archaea to humans. Annu Rev Cell Dev Biol 16:365–392. 11031241 10.1146/annurev.cellbio.16.1.365

[24] Martinez A, Bradley AS, Waldbauer JR, Summons RE, DeLong EF (2007) Proteorhodopsin photosystem gene expression enables photophosphorylation in a heterologous host. Proc Natl Acad Sci USA 104:5590–5595. 17372221 10.1073/pnas.0611470104 PMC1838496

[25] Walter JM, Greenfield D, Bustamante C, Liphardt J (2007) Light–powering *Escherichia coli* with proteorhodopsin. Proc Natl Acad Sci USA 104:2408–2412. 17277079 10.1073/pnas.0611035104 PMC1892948

[26] Balashov SP, Lanyi JK (2007) Xanthorhodopsin: Proton pump with a carotenoid antenna. Cell Mol Life Sci 64:2323–2328. 17571211 10.1007/s00018-007-7167-y PMC11138451

[27] Mongodin EF, Nelson K, Daugherty S, Deboy R, Wister J, et al (2005) The genome of Salinibacter ruber: convergence and gene exchange among hyperhalophilic bacteria and archaea. Proc Natl Acad Sci USA 102:18147–18152. 16330755 10.1073/pnas.0509073102 PMC1312414

[28] Hoff WD, Jung KH, Spudich JL (1997) Molecular mechanism of photosignaling by archaeal sensory rhodopsins. Annu Rev Biophys Biomol Struct 26:223–258. 9241419 10.1146/annurev.biophys.26.1.223

[29] Kunio I, Tohru U, Izumi K, Tomomi K-I, Yasuo S, et al (1999) Evolution of the archaeal rhodopsins evolution rate changes by gene duplication and functional differentiation. J Mol Biol 285:163–174. 9878396 10.1006/jmbi.1998.2286

[30] Frassanito AM, Barsanti L, Passarelli V, Evangelista V, Gualtieri P (2010) A rhodopsin-like protein in *Cyanophora paradoxa*:gene sequence and protein immunolocalization. Cell Mol Life Sci 67:965–971. 20016996 10.1007/s00018-009-0225-x PMC11115890

[31] Gualtieri P, Pelosi P, Passarelli V, Barsanti L (1992) Identification of a rhodopsin photoreceptor in *Euglena gracilis* . Biochim Biophys Acta 1117:55–59. 1627593 10.1016/0304-4165(92)90162-n

[32] Laura B, Vincenzo P, Paola L, Patricial W, Dunlap JR, et al (1993) Effects of hydroxylamine digitonin and triton X-100 on photoreceptor (paraflagellar swelling) and photoreception of *Euglena gracilis* . Vision Res 33:2043–2050. 8266645 10.1016/0042-6989(93)90002-e

[33] Lin S, Zhang H, Zhuang Y, Tran B, Gill J (2010) Spliced leader-based metatranscriptomic analyses lead to recognition of hidden genomic features in dinoflagellates. Proc Natl Acad Sci USA 107:20033–20038. 21041634 10.1073/pnas.1007246107 PMC2993343

[34] Sineshchekov OA, Govorunova EG, Jung KH, Zauner S, Maier UG, et al (2005) Rhodopsin–mediated photoreception in cryptophyte flagellates. Biophys J 89:4310–4319. 16150961 10.1529/biophysj.105.070920 PMC1366995

[35] Tsunoda SP, Ewers D, Gazzarrini S, Moroni A, Gradmann D, et al (2006) H^+^-pumping rhodopsin from the marine alga *Acetabularia* . Biophys J 91:1471–1479. 16731558 10.1529/biophysj.106.086421 PMC1518632

[36] Georg N, Doris O, Markus F, Suneel K, Anna MM, et al (2002) Channelrhodopsin-1 a light-gated proton channel in green algae. Science, New Series 296:2395–2398. 10.1126/science.1072068 12089443

[37] Sineshchekov OA, Jung KH, Spudich JL (2002) Two rhodopsins mediate phototaxis to low- and high-intensity light in *Chlamydomonas reinhardtii* . Proc Natl Acad Sci USA 99:8689–8694. 12060707 10.1073/pnas.122243399 PMC124360

[38] Zhang H, Hou Y, Miranda L, Campbell DA, Sturm NR, et al (2007) Spliced leader RNA trans-splicing in dinoflagellates. Proc Natl Acad Sci USA 104:4618–4623. 17360573 10.1073/pnas.0700258104 PMC1838650

[39] Slamovits CH, Okamoto N, Burri L, James ER, Keeling PJ (2011) A bacterial proteorhodopsin proton pump in marine eukaryotes. Nat Commun 2:1–6. 10.1038/ncomms1188 21304512

[40] Lowe CD, Keeling PJ, Martin LE, Slamovits CH, Watts PC, et al (2010) Who is *Oxyrrhis marina*? Morphological and phylogenetic studies on an unusual dinoflagellate. J Plankton Res 33:555–567.

[41] Slamovits CH, Keeling PJ (2010) Contributions of Oxyrrhis marina to molecular biology, genomics and organelle evolution of dinoflagellates. J Plankton Res 33:591–602.

[42] Lowe CD, Montagnes DJS, Martin LE, Watts PC (2010) Patterns of genetic diversity in the marine heterotrophic flagellate *Oxyrrhis marina* (Alveolata:Dinophyceae). Protist 161:212–221. 20034851 10.1016/j.protis.2009.11.003

[43] Guo ZL, Zhang H, Liu S, Lin S (2013) Biology of the marine heterotrophic dinoflagellate *Oxyrrhis marina*:current status and future directions. Microorganisms 1:33–57. 27694763 10.3390/microorganisms1010033 PMC5029500

[44] Hartz AJ, Sherr BF, Sherr EB (2011) Photoresponse in the heterotrophic marine dinoflagellate *Oxyrrhis marina* . J Eukaryot Microbiol 58:171–177. 21332875 10.1111/j.1550-7408.2011.00529.x

[45] Zhang H, Finiguerra M, Dam HG, Huang Y, Xu D, et al (2013) An improved method for achieving high-quality RNA for copepod transcriptomic studies. J Exp Mar Biol Ecol 446:57–66.

[46] Moreno-Paz M, Parro V (2006) Amplification of low quantity bacterial RNA for microarray studies: time-course analysis of *Leptospirillum ferrooxidans* under nitrogen-fixing conditions. Environ Microbiol 8:1064–1073. 16689727 10.1111/j.1462-2920.2006.00998.x

[47] Lin S, Zhang H (2010) Dinoflagellate meta-transcriptomics enabled by spliced leader. In Hongkong, Ho CK, editors. Proceedings of 13^th^ International Conference on Harmful Alge, International Society for the Study of Harmful Algae, pp. 166–170.

[48] Zhang H, Zhuang Y, Gill J, Lin S (2013) Proof that dinoflagellate spliced leader (DinoSL) is a useful hook for fishing dinoflagellate transcripts from mixed microbial samples: *Symbiodinium kawagutii* as a case study. Protist 164:510–527. 23773861 10.1016/j.protis.2013.04.002

[49] Lin X, Zhang H, Huang B, Lin S (2011) Alkaline phosphatase gene sequence and transcriptional regulation by phosphate limitation in *Amphidinium carterae* (Dinophyceae). J Phycol 47:1110–1120. 27020193 10.1111/j.1529-8817.2011.01038.x

[50] Thompson JD, Higgins GD, Gibson TJ (1994) CLUSTALW: improving the sensitivity of progressive multiple sequence alignment through sequence weighting, positions-specific gappenalties and weight matrix choice. Nucleic Acids Res 22:4673–4680. 7984417 10.1093/nar/22.22.4673 PMC308517

[51] Guindon S, Gascuel O (2003) A simple, fast, and accurate algorithm to estimate large phylogenies by maximum likelihood. Syst Biol 52:696–704. 14530136 10.1080/10635150390235520

[52] Saitou N, Nei M (1987) The neighbor-joining method: a new method for reconstructing phylogenetic trees. Mol Biol Evol 4:406–425. 3447015 10.1093/oxfordjournals.molbev.a040454

[53] Huelsenbeck JP, Ronquist F (2001) MRBAYES: Bayesian inference of phylogenetic trees. Bioinformatics 17:754–755. 11524383 10.1093/bioinformatics/17.8.754

[54] Zhang H, Hou Y, Lin S (2006) Isolation and characterization of proliferating cell nuclear antigen from the dinoflagellate *Pfiesteria piscicida* . J Eukaryot Microbiol 53:142–150. 16579817 10.1111/j.1550-7408.2005.00085.x

[55] Zhang H, Zhuang Y, Gill J, Lin S (2013) Proof that dinoflagellate spliced leader (DinoSL) is a useful hook for fishing dinoflagellate transcripts from mixed microbial samples: *Symbiodinium kawagutii* as a case study. Protist 164:510–527. 23773861 10.1016/j.protis.2013.04.002

[56] Gomez-Consarnau L, Gonzalez JM, Coll-Llado M, Gourdon P, Pascher T, et al (2007) Light stimulates growth of proteorhodopsin-containing marine Flavobacteria. Nature 445:210–213. 17215843 10.1038/nature05381

[57] Wang Z, O'Shaughnessy TJ, Soto CM, Rahbar AM, Robertson KL, et al (2012) Function and regulation of *Vibrio campbellii* proteorhodopsin: acquired phototrophy in a classical organoheterotroph. PLoS ONE 7:1–8. 10.1371/journal.pone.0038749 PMC3380642 22741028

[58] Jeong HJ, Seong KA, Yoo YD, Kim TH, Kang NS, et al (2008) Feeding and grazing impact by small marine heterotrophic dinoflagellates on heterotrophic bacteria. J Eukaryot Microbiol 55:271–288. 18681841 10.1111/j.1550-7408.2008.00336.x

[59] Skovgaard A (1996) Mixotrophy in *Fragilidium subglobosum* (Dinophyceae): growth and grazing responses as functions of light intensity. Mar Ecol Prog Ser 143:247–253.

[60] Caron DA, Sanders RW, Lim EL, Marrasé C, Amaral L, et al (1993) Light-dependent phagotrophy in the freshwater mixotrophic chrysophyte *Dinobryon cylindricum* . Microb Ecol 25:93–111. 24189708 10.1007/BF00182132

[61] Li A, Stoecker DK, Coats DW (2000) Mixotrophy in *Gyrodinium galatheanum* (DINOPHYCEAE): grazing responses to light intensity and inorganic nutrients. J Phycol 36:33–45.

[62] Feinstein TN, Traslavina R, Sun M, Lin S (2002) Effects of light on photosynthesis, grazing, and population dynamics of the heterotrophic dinoflagellate *Pfiesteria Piscicida* (Dinophyceae). J Phycol 38:659–669.

[63] Gomez-Consarnau L, Akram N, Lindell K, Pedersen A, Neutze R, et al (2010) Proteorhodopsin phototrophy promotes survival of marine bacteria during starvation. PLoS Biol 8:1–10. 10.1371/journal.pbio.1000358 PMC2860489 20436956

[64] Marchetti A, Schruth DM, Durkin CA, Parker MS, Kodner RB, et al (2011) Comparative metatranscriptomics identifies molecular bases for the physiological responses of phytoplankton to varying iron availability. Proc Natl Acad Sci USA 109:E317–E325. 10.1073/pnas.1118408109 PMC3277525 22308424

[65] Feng S, Powell SM, Wilson R, Bowman JP (2013) Light-stimulated growth of proteorhodopsin-bearing sea-ice psychrophile *Psychroflexus torquis* is salinity dependent. ISME J 7:2206–2213. 23788334 10.1038/ismej.2013.97 PMC3806269

[66] Kuo RC, Zhang H, Zhuang Y, Hannick L, Lin S (2013) Transcriptomic study reveals widespread spliced leader *trans*-splicing, short 5'-UTRs and potential complex carbon fixation mechanisms in the euglenoid Alga *Eutreptiella sp* . PLoS One 8:1–12. 10.1371/journal.pone.0060826 PMC3621762 23585853

[67] Zhang H, Bhattacharya D, Lin S (2005) Phylogeny of Dinoflagellates Based on Mitochondrial Cytochrome B and Nuclear Small Subunit Rdna Sequence Comparisons. J Phycol 41:411–420.

