# Supplementary figures and images for: Light-Promoted Rhodopsin Expression and Starvation Survival in the Marine Dinoflagellate Oxyrrhis marina
Source: PLoS One. 2014 Dec 15;9(12):e114941. doi: 10.1371/journal.pone.0114941 (PMC4266641; doi:10.1371/journal.pone.0114941)

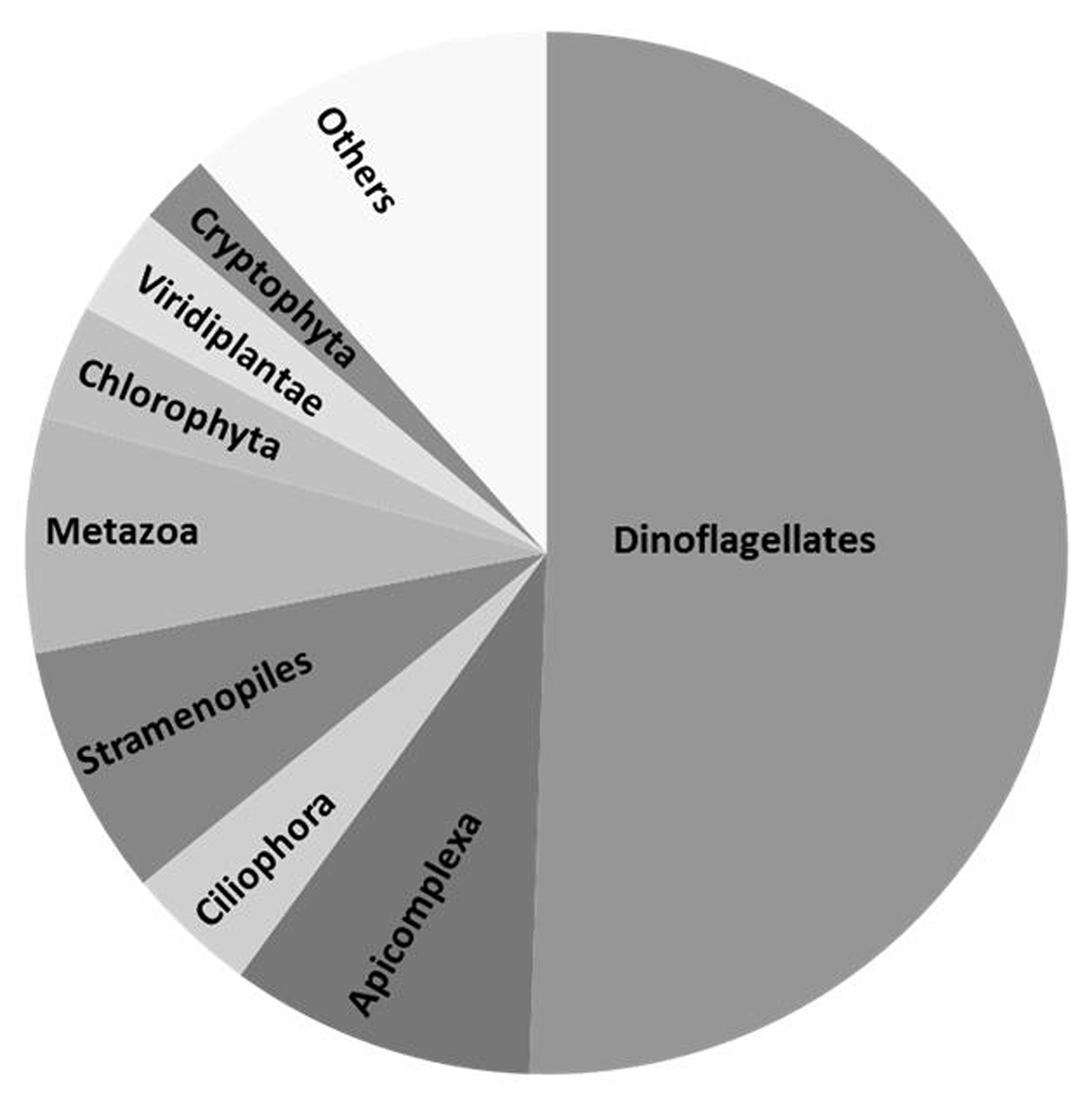

Supplement: S1 Figure — Summarized Top-Hit lineage distribution of the cDNAs recovered from our O . marina transcriptomic data. The majority of the sequences either hit dinoflagellates or their alveolate relatives (apicomplexans and ciliates) or had no matches (others) in GenBank database. (TIF) [file pone.0114941.s001.tif]

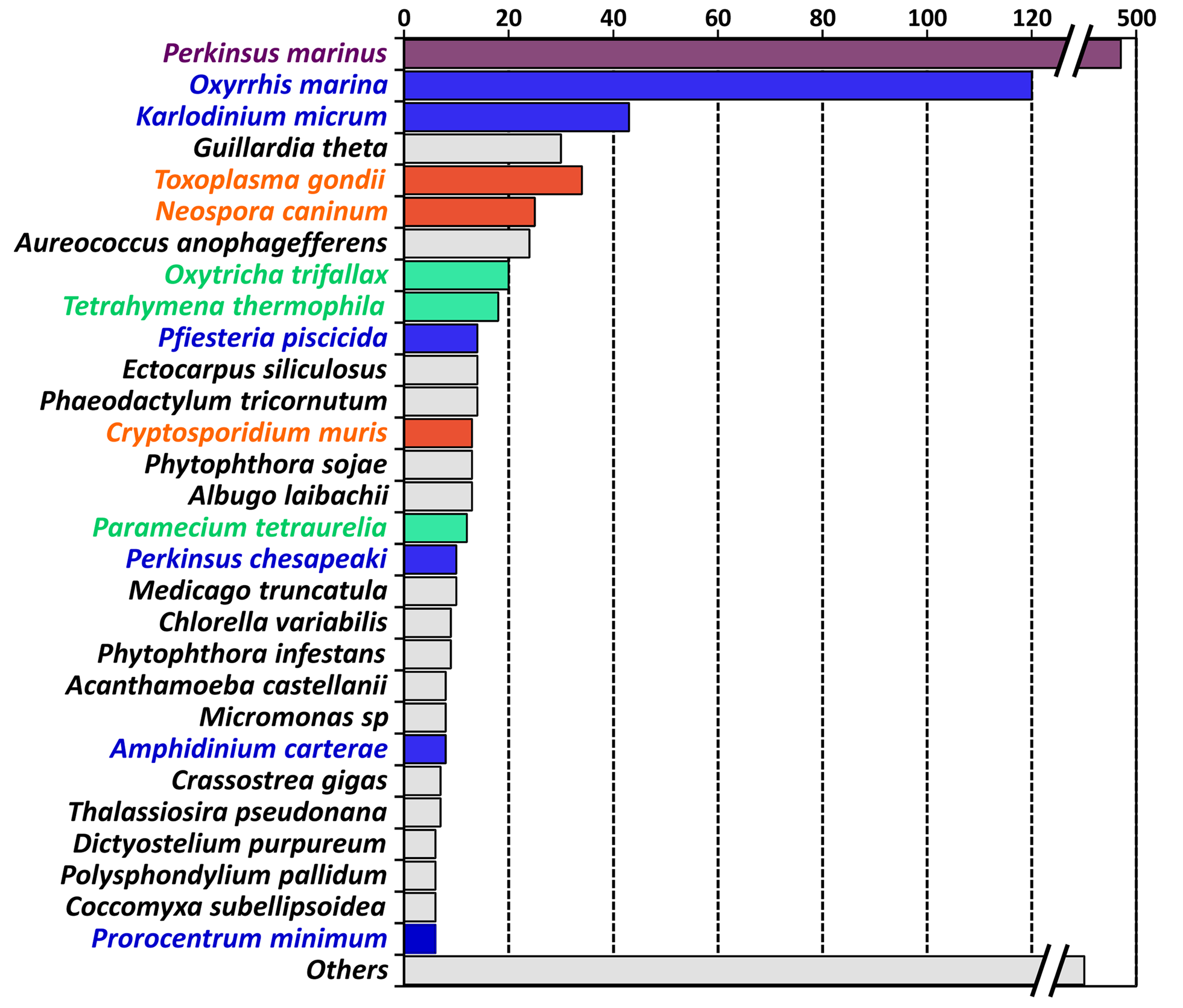

Supplement: S2 Figure — Detailed Top-Hit species distribution of the cDNAs recovered from our O . marina transcriptomic data. The majority of the sequences hit dinoflagellates (blue) or their alveolate relatives, apicomplexans (orange), Perkinsus marinus (purple) and ciliates (green), and a small fraction hit unknown or nondinoflagellate organisms (gray). (TIF) [file pone.0114941.s002.tif]

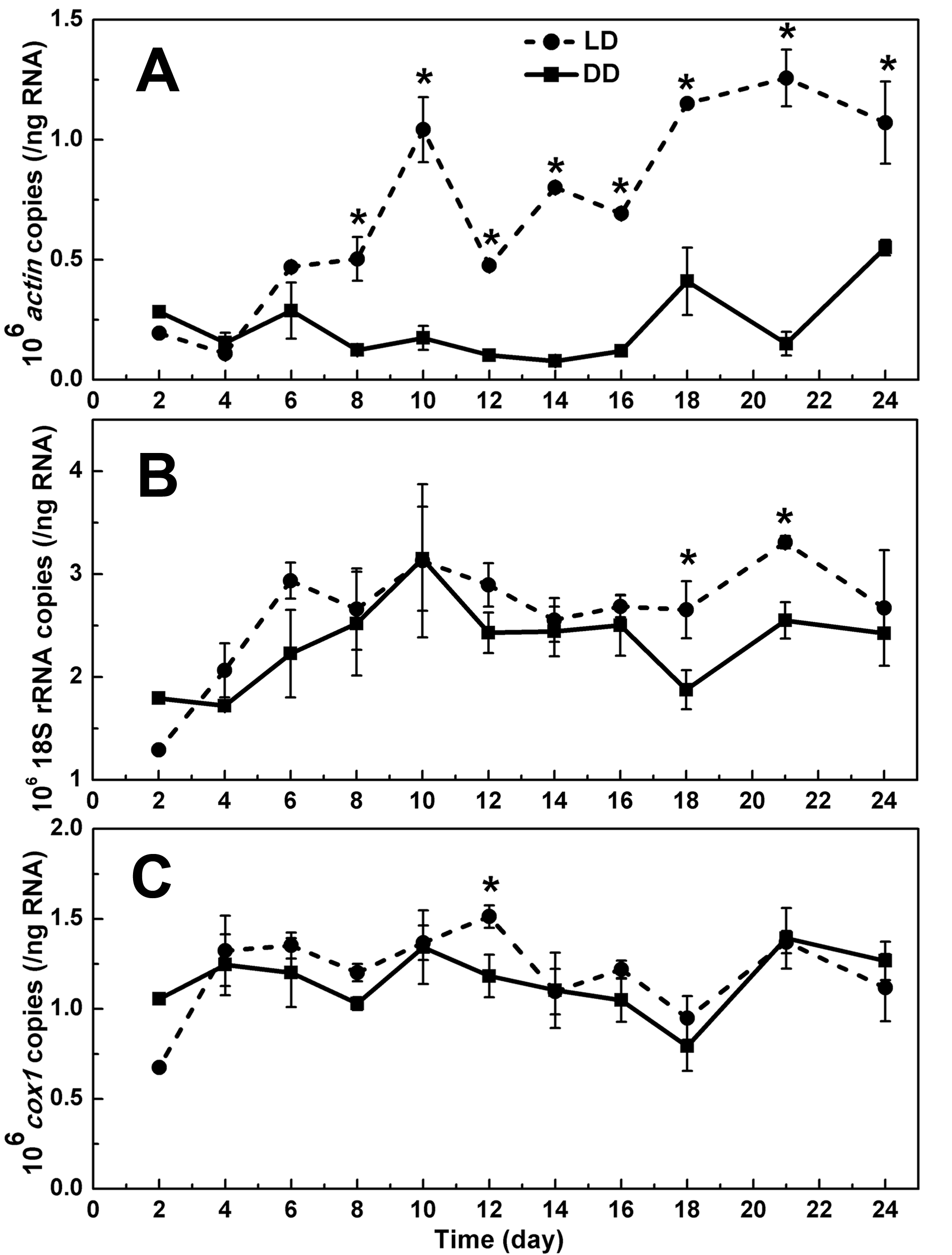

Supplement: S4 Figure — Expression levels of three potentially house-keeping genes as normalized to the amount of total RNA. LD, cultures grown under light: dark cycle; DD, cultures grown under continuous darkness; error bars, standard deviation. (A) actin; (B) 18S rRNA; (C) cox1. Significant differences between light and dark group were marked with “*”. (TIF) [file pone.0114941.s004.tif]
