# Supplementary material for: Light-Promoted Rhodopsin Expression and Starvation Survival in the Marine Dinoflagellate Oxyrrhis marina
Source: PLoS One. 2014 Dec 15;9(12):e114941. doi: 10.1371/journal.pone.0114941 (PMC4266641; doi:10.1371/journal.pone.0114941)
Supplement: S1 Table — Alignment of PR and SR types of O. marina rhodopsin to indicate similarity and difference. Asterisks depict identical amino acid residues; dots depict positions where residues are chemically similar, with positions dominated by one residue indicated by double dots. In yellow shade are regions conserved in the PR type but absent in the SR type. In red letters are residues that compose retinal pocket, which are all conserved in the PR type but partially different in the SR type. Triangle indicates position where proton donor (green) and receiver (blue) are expected, which are also conserved in the PR type but not so conserved in the SR type. (DOC) [file pone.0114941.s005.doc]

| Oxyma_ABV22426.1 | MAPLTGDFSYGEWNAVYNALSFGIAAMGSATVFFWLQLGNVS----KNYR |
| --- | --- |
| Oxyma_ADY17807.1 | MAPLTGDFSYGEWNAVYNALSFGIAAMGSATVFFWLQLGNVS----KNYR |
| Oxyma_ABV22428.1 | MAPLTGDFSYGEWNAVYNALSFGIAAMGSATVFFWLQLGNVS----KNYR |
| Oxyma_ABV22432.1 | MAPLTGDFSYGEWNAVYNALSFGIAAMGSATVFFWLQLGNVT----KNYR |
| Oxyma_ADY17808.1 | MAPLTGDFSYGEWNAVYNALSFGIAAMGSATVFFWLQLGNVT----KSYR |
| Oxyma_ADY17811.1 | MAPLAGDFSYGEWNAVYNALSFGIAAMGSATVFFWLQLPNVT----RSYR |
| Oxyma_ADY17809.1 | MAPLAGDFTYGQWSAVYNALSFGIAAMGSATVFFWLQLPNVT----KSYR |
| Oxyma_ABV22427.1 | MAPLAQDWTYAEWSAVYNALSFGIAGMGSATIFFWLQLPNVT----KNYR |
| Oxyma_ADY17806.1 | MAPLAQDWTYAEWSAVYNALSFGIAGMGSATIFFWLQLPNVT----KNYR |
| Oxyma_ADY17810.1 | ----MGVHTWSRSEAGSQETLFAIFVIFAIAFLWVLLLSQQS-----KSK |
| SR1_KF651052 | ----MGVHTWSRSEAGSQETLFAIFVIFAIAFLWVLLLSQQS-----KSK |
| Oxyma_AEA49880.1 | ------MFALTTCEKATSWVVFAIFVISSLIFMFRNSAADSSYEGGAKSQ |
| SR2a_KF651053 | ---------MATCEVVTLWICAALFLLAALVFMFKERS-------YPKAK |
| SR2b_KF651054 | ---------MATCEVVTLWICAALFLLAALVFMFKERS-------YPKAK |
| SR3_KF651055 | -------MTVSNCTVSTLWVACGVFTVSALIFLFKERS-------HVKAK |
|  | .: : : .:: . : |
| Oxyma_ABV22426.1 | TALTITGIVTWIATYHYFRIFNSWVEAFEVN---EVGGAYAVKVSGTPFN |
| Oxyma_ADY17807.1 | TALTITGIVTWIATYHYFRIFNSWVEAFEVN---EVGGAYAVKVSGTPFN |
| Oxyma_ABV22428.1 | TALTITGIVTWIATYHYFRIFNSWVEAFEVN---EVGGAYAVKVSGTPFN |
| Oxyma_ABV22432.1 | TALTITGIVTWIATYHYFRIFNSWVEAFEVN---EVGGAYSVKVSGTPFN |
| Oxyma_ADY17808.1 | TALTITGIVTWIATYHYFRIFNSWVEAFEVN---EVGGAYSVKVSGTPFN |
| Oxyma_ADY17811.1 | TALTITGIVTWIATYHYFRIFNSWVEAFEVQ---EYHGAYLVKVSGTPFN |
| Oxyma_ADY17809.1 | TALTITGIVTWIATYHYFRIFNSWVAAFEVQ---QAGGDYAVSVSGTPFN |
| Oxyma_ABV22427.1 | TALTITGIVTLIATYHYFRIFNSWVAAFNVGL--GVNGAYEVTVSGTPFN |
| Oxyma_ADY17806.1 | TALTITGIVTLIATYHYFRIFNSWVAAFNVGL--GVNGAYEVTVSGTPFN |
| Oxyma_ADY17810.1 | KYYYVSAAILAVAACAYYFMAWG----------YGILDNGQAWHTDGKHL |
| SR1_KF651052 | KYYYVSAAILAVAACAYYFMAWG----------YGILDNGQAWHTDGKHL |
| Oxyma_AEA49880.1 | RYRLVTASITIIAATFYFFMAQG----------YGVATS------VNHEF |
| SR2a_KF651053 | QFNYTTALMNFIAATAYFFMALAMEDPN-VAFPGISLGTIPNGAGGFRKF |
| SR2b_KF651054 | QFNYTTALMNFIAATAYFFMALAMEDPN-VAFPGISLGTIPNGAGGFRKF |
| SR3_KF651055 | QYNFVTALMNFVAAAAYFLMALQPDPTTPEAFFGVVVGSAPDGQGSFRDL |
|  | :. : :*: *: : |
|  |  |
| Oxyma_ABV22426.1 | DAYRYVDWLLTVPLLLIELILVMKLPAGETAALSTKLGVASAVMVALGYP |
| Oxyma_ADY17807.1 | DAYRYVDWLLTVPLLLIELILVMKLPAGETAALSTKLGVASAVMVALGYP |
| Oxyma_ABV22428.1 | DAYRYVDWLLTVPLLLIELILVMKLPAGETAALSTKLGVASAVMVALGYP |
| Oxyma_ABV22432.1 | DAYRYVDWLLTVPLLLIELILVMKLPAGETAALSTKLGVASAVMVALGYP |
| Oxyma_ADY17808.1 | DAYRYVDWLLTVPLLLIELILVMKLPAGETAALSTKLGVASAVMVALGYP |
| Oxyma_ADY17811.1 | DAYRYVDWLLTVPLLLIELILVMKLPSGETAAMGTKLGLASAVMVALGYP |
| Oxyma_ADY17809.1 | DAYRYVDWLLTVPLLLIELILVMKLPAGETASMGTKLGLASAVMVALGYP |
| Oxyma_ABV22427.1 | DAYRYVDWLLTVPLLLVELILVMKLPAKETVCLAWTLGIASAVTVALGYP |
| Oxyma_ADY17806.1 | DAYRYVDWLLTVPLLLVELILVMKLPAKETVCLAWTLGIASAVMVALGYP |
| Oxyma_ADY17810.1 | FWLRYLDWLITTPLLLLDLALLAGLDFWET----GFIILMDMLMITAGYI |
| SR1_KF651052 | FWLRYLDWLITTPLLLLDLALLAGLDFWET----GFIILMDMLMITAGYI |
| Oxyma_AEA49880.1 | WWLRYLDWLITTPLLLLDLALIAGIDVWDT----FALLVADVLMITVGFV |
| SR2a_KF651053 | FWMRYLSWFLTTPLILLDLGMLSGFDFWDN----FLMIALDLCMIAAGYV |
| SR2b_KF651054 | FWMRYLSWFLTTPLILLDLGMLSGFDFWDN----FLMIALDLCMIAAGYV |
| SR3_KF651055 | YWLRYLSWAATTPLILLDMGMLAGFDFWEN----FLLIVLDILMIACGYV |
|  | **:.* *.**:*::: :: : :. : . :: *: |
| Oxyma_ABV22426.1 | GEIQENLAVRWFWWALAMIPFAYVVFSLLVGLGAATAKQPESV--AGLVS |
| Oxyma_ADY17807.1 | GEIQENLAVRWFWWALAMIPFAYVVFSLLVGLGAATAKQPESV--AGLVS |
| Oxyma_ABV22428.1 | GEIQENLAVRWFWWALAMIPFAYVVFSLLVGLGAATAKQPESV--AGLVS |
| Oxyma_ABV22432.1 | GEIQENLAVRWFWWALAMIPFAYVVFSLLVGLGAATAKQPESV--AGLVS |
| Oxyma_ADY17808.1 | GEIQENLAVRWFWWALAMIPFAYVVFSLLVGLGAATAKQPESV--AGLVS |
| Oxyma_ADY17811.1 | GEIQDNLAVRWGWWALAMIPFFYVVYSLLSGLGEATARQPESV--GGLVS |
| Oxyma_ADY17809.1 | GEIQENLAVRWFWWALAMIPFFYVVYSLLAGLGEATAKQPESV--SGLVS |
| Oxyma_ABV22427.1 | GEIQDDLSVRWFWWACAMVPFVYVVGTLVVGLGAATAKQPEGV--VDLVS |
| Oxyma_ADY17806.1 | GEIQDDLSVRWFWWACAMVPFVYVVGTLVVGLGAATAKQPEGV--VDLVS |
| Oxyma_ADY17810.1 | GASTEQ--FVWQGFGVSMVFFILVLGYLGDGVLALDEDSKNTG----TAR |
| SR1_KF651052 | GASTEQ--FVWQGFGVSMVFFILVLGYLGDGVLALDEDSKNTG----TAR |
| Oxyma_AEA49880.1 | AGNPDYG-HTWECFAVSMAFFLLTLYIIGEGMLEHADDPNTSEEKERLMK |
| SR2a_KF651053 | G-AGEHA-LEWQAFSLSFFFFVALMFFLTTGVLQKANEEANDD-RAARMT |
| SR2b_KF651054 | G-AGEHA-LEWQAFSLSFFFFVALMFFLTTGVLQKANEEANDD-RAARMT |
| SR3_KF651055 | G-AAEDA-LEFKAFAPGMVLFVIVMFRLANGVQLAAQTDDSDD-KAAKMG |
|  | . : : :. .: * : : *: |
| Oxyma_ABV22426.1 | AARYLTAVSWLTYPFVYIIKNVGLAGP---TATMYEQIGYSVADVMAKAV |
| Oxyma_ADY17807.1 | AARYLTAVSWLTYPFVYIIKNVGLAGP---TATMYEQIGYSVADVMAKAV |
| Oxyma_ABV22428.1 | AARYLTAVSWLTYPFVYIIKNVGLAGP---TATMYEQIGYSVADVMAKAV |
| Oxyma_ABV22432.1 | AARYLTAVSWLTYPFVYIIKNVGLAGP---TATMYEQIGYSVADVMAKAV |
| Oxyma_ADY17808.1 | AARYLTAVSWLTYPFVYIIKNVGLAGP---TATMYEQIGYSVADVMA--- |
| Oxyma_ADY17811.1 | AARYLTAVSWLTYPFVYIIKNVG--------------------------- |
| Oxyma_ADY17809.1 | AARYLTAVSWLTYPFVYIIKNIGLAGP---TATMCEQIGYSIADVVAKAV |
| Oxyma_ABV22427.1 | AARYLTVVSWLTYPFVYIVKNIGLAGS---TATMYEQIGYSAADVTAKAV |
| Oxyma_ADY17806.1 | AARYLTVVSWLTYPFVYIVKNIGLAGS---TATMYEQIGYSAADVTAKAV |
| Oxyma_ADY17810.1 | NLFWLTVLIWCTYPLYFVLEHTMGLST------FQEILCYGISDVLAKVV |
| SR1_KF651052 | NLFWLTVLIWCTYPLYFVLEHTMGLST------FQEILCYGISDVLAKVV |
| Oxyma_AEA49880.1 | TLLWLTVLIWCTYPLYFVLEYSNVLNNNSTNHRCTLVLLYGLSDVVAKSV |
| SR2a_KF651053 | TLLWLTVIVWCTYPVYFVLVQRNVLSQ------CAEVVVFAISDVLSKAV |
| SR2b_KF651054 | TLLWLTVIVWCTYPVYFVLVQRNVLSQ------CAEVVVFAISDVLSKAV |
| SR3_KF651055 | LLLGITVVTWACYPLFYLLCQQNLISE------CAEVISFAVLDVVSKAV |
|  | :*.: * **. ::: |
| Oxyma_ABV22426.1 | FGVLIWAIANEKSRLEGEGKLLR------------- |
| Oxyma_ADY17807.1 | FGVLIWAIANEKSRLEGEGKLLR------------- |
| Oxyma_ABV22428.1 | FGVLIWAIANEKSRLEGEGKLLR------------- |
| Oxyma_ABV22432.1 | FGVLIWAIANEKSRLESEGKLLW------------- |
| Oxyma_ADY17808.1 | ------------------------------------ |
| Oxyma_ADY17811.1 | ------------------------------------ |
| Oxyma_ADY17809.1 | FGVLIWAIAAEKSRLEELSKPGRR------------ |
| Oxyma_ABV22427.1 | FGVLIWAIANAKSRLEEEGKLRA------------- |
| Oxyma_ADY17806.1 | FGVLIWAIANAKSRLEEEGKLRA------------- |
| Oxyma_ADY17810.1 | FALVLVYNFDDDDEPAVYQQQMVVMQQQQPMVTTIG |
| SR1_KF651052 | FALVLVYNFDDDDEPAVYQQQMVVMQQQQPMVTTVA |
| Oxyma_AEA49880.1 | FGFILLMN---DDLLHVAMPTGVVEQIP-------- |
| SR2a_KF651053 | FGILLLADEDLLSSLQDGGAGQIAMTTQPQVIA--- |
| SR2b_KF651054 | FGILLLADEDLLSSLQDGGAGQIAMTTQPQVIA--- |
| SR3_KF651055 | FGFVLLRDEDML----------VDSTEIPQVVGT-- |
